# Simultaneous Lipid and Carotenoid Production via *Rhodotorula paludigena* CM33 Using Crude Glycerol as the Main Substrate: Pilot-Scale Experiments

**DOI:** 10.3390/ijms242417192

**Published:** 2023-12-06

**Authors:** Cheeranan Sriphuttha, Pailin Boontawan, Pasama Boonyanan, Mariena Ketudat-Cairns, Apichat Boontawan

**Affiliations:** 1 School of Biotechnology, Institute of Agricultural Technology, Suranaree University of Technology, 111 University Avenue, Muang District, Nakhon Ratchasima 30000, Thailand; cheeranan.nicky@gmail.com (C.S.); pailinboontawan@gmail.com (P.B.); ketudat@sut.ac.th (M.K.-C.); 2 The Center for Scientific and Technological Equipment, Suranaree University of Technology, 111 University Avenue, Muang District, Nakhon Ratchasima 30000, Thailand; pasama.boonyanan@sut.ac.th; 3 Center of Excellent in Agricultural Product Innovation, Suranaree University of Technology, 111 University Avenue, Muang District, Nakhon Ratchasima 30000, Thailand

**Keywords:** oleaginous yeast, crude glycerol, *R. paludigena* CM33, bio-lipids, carotenoids

## Abstract

*Rhodotorula paludigena* CM33 is an oleaginous yeast that has been demonstrated to accumulate substantial quantities of intracellular lipids and carotenoids. In this study, crude glycerol, a by-product of biodiesel production, was used as a carbon source to enhance the accumulation of lipids and carotenoids in the cells. The culture conditions were first optimized using response surface methodology, which revealed that the carotenoid concentration and lipid content improved when the concentration of crude glycerol was 40 g/L. Different fermentation conditions were also investigated: batch, repeated-batch, and fed-batch conditions in a 500 L fermenter. For fed-batch fermentation, the maximum concentrations of biomass, lipids, and carotenoids obtained were 46.32 g/L, 37.65%, and 713.80 mg/L, respectively. A chemical-free carotenoid extraction method was also optimized using high-pressure homogenization and a microfluidizer device. The carotenoids were found to be mostly beta-carotene, which was confirmed by HPLC (high pressure liquid chromatography), LC-MS (liquid chromatography-mass spectrometry), and NMR (nuclear magnetic resonance). The results of this study indicate that crude glycerol can be used as a substrate to produce carotenoids, resulting in enhanced value of this biodiesel by-product.

## 1. Introduction

The surge in energy demand in recent times has led to increasing interest in novel alternative feedstocks, such as bio-lipids, which are generated by microorganisms and have high energy density and eco-friendly traits [1]. Microorganisms such as bacteria, algae, yeast, and fungi are capable of accumulating intracellular lipids, with yields exceeding 20% of their dry weight [2]. In addition, microbial lipids have drawn much attention for many applications, including industrial biofuel and oleochemical applications. Furthermore, the potential of oleaginous fungi and yeast to generate lipids not found in the animal or plant kingdom has sparked curiosity. Oleaginous molds accumulate unsaturated compounds more than yeasts. The genera of oleaginous yeast include *Rhodotorula*, *Rhodosporidium*, *Yarrowia*, *Candida*, *Trichosporon*, and *Cryptococcus* [3]. Oleaginous yeasts accumulate small fractions of glycolipids, phospholipids, and free fatty acids. The fatty acid profiles of oleaginous yeasts mainly consist of C16 and C18 fatty acids, such as palmitic acid (C16:0), stearic acid (C18:0), oleic acid (C18:1), and linoleic acid (C18:2), which is a composition comparable to that of plant lipids [4]. To exploit the potential of oleaginous yeast as microbial cell factories, various variables must be considered, including lipid production, fatty acid composition, and the utilization of inexpensive materials as nutrition sources [5]. Oleaginous yeasts have great potential to function as a sustainable feedstock for the oleochemical industry due to their high lipid content and rapid growth rate. They can also be cultivated using various low-cost organic agro-industrial residues (OAIRs), such as crude glycerol [6,7,8], molasses [9], lignocellulose [10,11,12], wastewater [13,14,15], and municipal waste sludge [16]. Apart from lipids, oil-soluble substances, especially carotenoids, are of interest due to their high nutritional values. Carotenoids are natural pigments used in many industries, such as the food supplement, pharmaceutical, cosmetics, and chemical industries. The biochemistry of de novo carotenoid synthesis has been intensively reviewed [17]. The pigments are usually found in fruits, vegetables, microalgae, mold, bacteria, and yeast. The global carotenoid market reached USD 1.5 billion in 2019, and it is estimated to reach USD 2.0 billion by 2026 [18]. However, carotenoid production from plants has some disadvantages, such as high production costs involving extensive requirements like irrigation, fertilizer, land, labor, and time. Furthermore, it is significantly influenced by the season [19].

Red yeasts have been identified as potential carotenoid producers among carotenoid-producing microorganisms. They have the ability to grow quickly in several substrates, thereby significantly reducing the production time that would be required at an industrial scale [20]. Currently, the fermentation of microbial carotenoids is being extensively researched using different red yeasts, including *Phaffia rhodozyma* [21], *Sporidiobolus pararoseus* [11], *Rhodosporidium kratochvilovae* [22], *Rhodosporidium toruloides* [8], *Rhodotorula* sp. [23], *Rhodotorula glutinis* [24,25], *Rhodotorula mucilaginosa* [26,27], and *Rhodotorula paludigina* CM33 [4]. The yeast *R. paludigina* CM33 has been shown to have the capability to utilize various carbon sources, including glucose, sucrose, xylose, and glycerol [4]. For further process development, employing readily available and cheap agro-industrial substrates would lower operational costs as well as alleviate environmental contamination. Among the tested substrates, crude glycerol has been of interest because it is a by-product of the biodiesel production process. The economic value of this industry could be increased by gaining more triglyceride feedstock for biodiesel and oleochemicals, as well as producing carotenoids for high-value applications. The bioprocess of carotenoid production involves upstream and downstream processing. For upstream processing, optimizing the yeast’s growth and ability to accumulate high levels of carotenoids is of interest. Genetic modification to improve carotenoid accumulation has been investigated using many techniques, such as gene insertion [28], light irradiation [24,29], and chemical treatments [30]. However, there are health concerns regarding the safety of genetically modified foods. As a result, most of the research has focused on media optimization, culture conditions, and fermentation modes to produce natural carotenoids [1,13]. Regarding downstream processes, several techniques have been investigated to enhance the separation and purification of carotenoids. Extractions by non-chemical methods have been studied, such as high-pressure homogenization [31], ultrasound under pressure [32], and pulsed electric fields [33]. The final process for high-purity production has been carried out by chromatographic separation [34].

In this study, process development was investigated using media formulations based on crude glycerol concentrations, salts, and nitrogen sources. The effects of cultivation conditions on cell growth, carotenoid concentration, and lipid yield were explored using response surface methodology (RSM). The optimized conditions were subsequently applied to a 500 L stirred tank bioreactor for batch, repeated-batch, and fed-batch fermentations. After harvesting the cells, carotenoids were extracted and purified using a microfluidizer, followed by preparative liquid chromatography. The aim of this study was to develop a feasible downstream process for potential oleaginous yeasts and to pave the way for the commercial production of carotenoids and lipids from oleaginous yeast using agro-industrial by-products.

## 2. Results and Discussion

### 2.1. Characterization of Crude Glycerol

The chemical composition of crude glycerol used in this study was found to contain 58% glycerol and a pH value of 2.38. The element analysis of the crude glycerol showed that it contained 25.91% carbon, 7.44% hydrogen, less than 1% nitrogen, 4.14% sulfur, and 11,769 ppm of sodium, as shown in Table 1. The chemical composition of crude glycerol depends on several factors, such as the downstream processing methods, type of catalyst used, and transesterification efficiency. Crude glycerol is often purified using chemicals during the biodiesel production process, resulting in a pH value as low as 1 [35]. These findings align with previous reports, which stated that crude glycerol had a carbon content of 24.3%. Additionally, carbon content ranging from 24% to 37% was detected in seven different types of crude glycerol [36,37]. The low nitrogen value obtained in this study (<1%) also shows good agreement with the findings of previous research [37]. The sample of crude glycerol used in this study had a high sodium concentration (11,769 ppm) because of the use of basic catalysts and the harvesting of the glycerol phase with 12.5 M NaOH during biodiesel production [35]. After pretreatment, the glycerol concentration increased to approximately 75%. Previous studies have employed crude glycerol as a source of carbon to cultivate oleaginous yeast for the production of lipids and carotenoids [38]. Hence, the crude glycerol in this study can be used as a carbon source to culture *R. paludigena* CM33. However, it was observed that the crude glycerol had a low nitrogen concentration. Therefore, yeast extract and (NH_4_)_2_SO_4_ were added and optimized through RSM. In addition, before the experiment, the crude glycerol was pretreated each time, and its concentration was analyzed using HPLC.

### 2.2. Media Optimization for Carotenoid and Lipid Production in R. paludigena CM33

**Figure 1:**
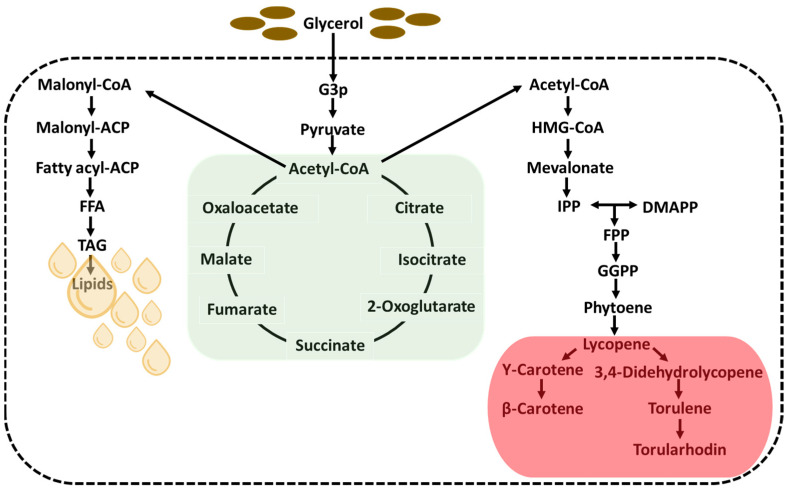
Biosynthetic pathways of carotenoids and lipids in *Rhodotorula* sp. using glycerol as carbon source [41,44].

The carotenoid and lipid production of yeast is known to require a medium with abundant carbon and limited nitrogen sources [7]. The excess carbon source is consistently utilized to generate and store lipids [39]. In addition, a reduction in carotenoid production can occur due to a lower concentration of nitrogen in the medium [40]. The synthesis of lipids and carotenoids in *R. paludigena* CM33 is inter-related and is demonstrated through the metabolic pathway in Figure 1. The intensity of carotenoid and lipid biosynthesis experience indirect influences by the conversion of glycerol or glucose to pyruvate (pyruvate biosynthesis) and then to acetyl-CoA (TCA cycle), along with other metabolic pathways linked to carotenoid and lipid biosynthesis [41]. Therefore, the main factors considered as independent variables in this study were crude glycerol (X_1_), yeast extract (X_2_), and (NH_4_)_2_SO_4_ (X_3_). These factors were selected for optimization of the lipid content and carotenoid production. The combined effects of three independent variables on the lipid content and carotenoid concentration were determined using a central composite design and RSM. The results were subjected to an analysis of variance (ANOVA), as shown in Table 2. Experimental results showed that glycerol (X_1_) has positive effects on the resulting responses (*p* < 0.05). The model F-values of 104.44 for lipids and 216.78 for carotenoids imply that the model is extremely significant, with a *p*-value less than 0.05. The lack of insignificance in the lack-of-fit test indicated the accuracy of the model in predicting lipid and carotenoid production. The model successfully predicted the occurrence of variance in the test and fitted the experimental data, as indicated by the obtained *p*-value of <0.05 [42,43]. The coefficient of determination was R^2^ = 0.9947 for lipids and R^2^ = 0.9974 for carotenoids. The adjusted R^2^ value was 0.9852 for lipids and 0.9928 for carotenoids (Table 2). The collected data were subjected to linear regression analysis using the software Design Expert 13, and a quadratic polynomial model was used to express the relationship between the variables. The model is expressed in Equations (1) and (2), where Y represents the lipid and carotenoids, and X_1_, X_2_, and X_3_ represent the variables of crude glycerol, yeast extract, and (NH_4_)_2_SO_4_, respectively.

Y_carotenoid_ = −581.99 + (21.03 X_1_) + (1270.67 X_2_) + (160.94 X_3_) − (2.20 (X_1_ × X_2_)) + (0.43 (X_1_ × X_3_)) − (53.33 (X_2_ × X_3_)) − (0.23 X_1_^2^) − (880.00 X_2_^2^) − (255.56 X_3_^2^) (1)

Y_lipid_ = −27.75 + (2.61 X_1_) − (5.66 X_2_) + (50.50 X_3_) − (0.62 (X_1_ × X_2_)) − (0.22 (X_1_ × X_3_)) + (54.40 (X_2_ × X_3_)) − (0.03 X_1_^2^) + (28.37 X_2_^2^) − (88.52 X_3_^2^) (2)

**Figure 2:**
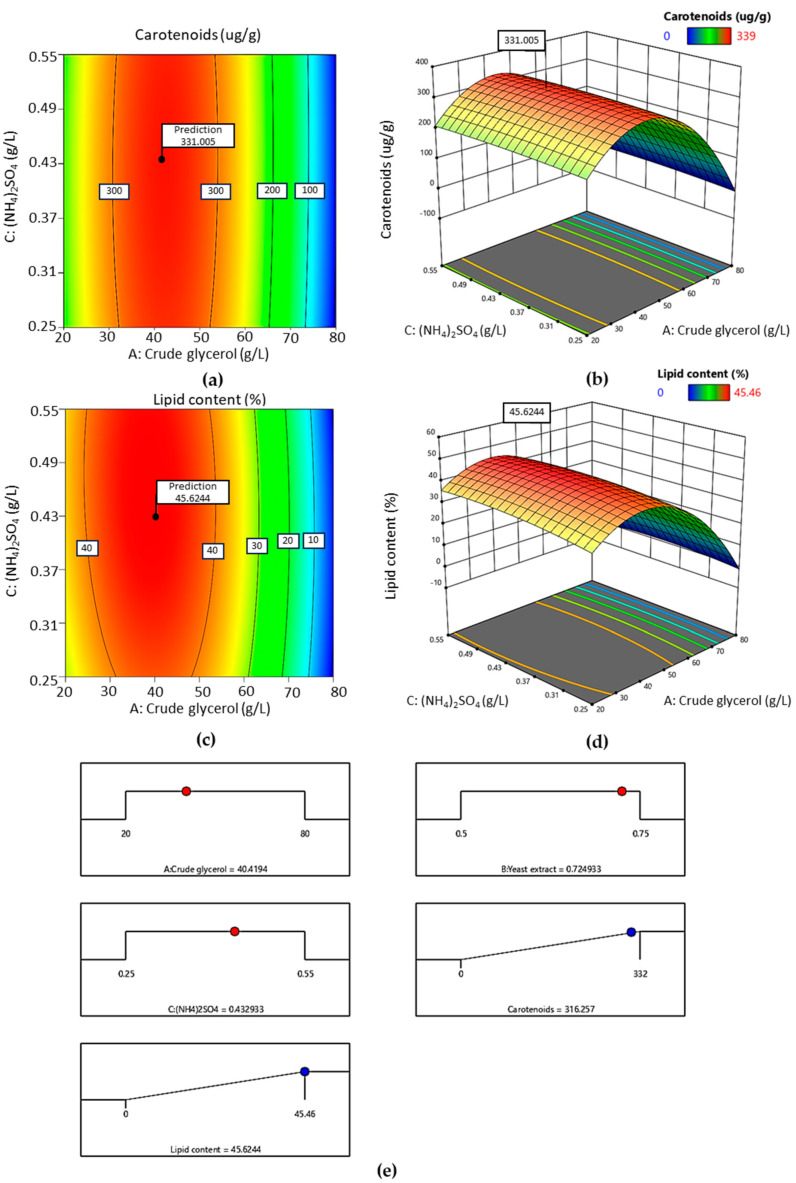
The effect of different significant variables on the response surface contour plot was evaluated using response surface methodology for carotenoids and lipid production (**a**,**c**). The RSM 3D surface plots obtained using Design Expert software illustrate the effects of different significant variables on carotenoids and lipid production by *R. paludigena* CM33 (**b**,**d**). The optimized parameters predicted by RSM were 40 g/L of crude glycerol, 0.72 g/L of yeast extract, and 0.43 g/L of (NH_4_)_2_SO_4_ concentration (**e**).

The regression equations of significant parameters for lipid content and carotenoid concentration were graphically represented in RSM contour plots and 3D surface plots generated by Design Expert 13. The relationship and effect between different significant variables regarding lipid content and carotenoid concentration are shown in Figure 2a–d. The contour and 3D surface graphs were plotted with a combination of parameters and a fixed yeast extract concentration, while the remaining parameters were kept constant at the maximum level. The figures show that the maximum lipid content and carotenoid concentration occurred when the crude glycerol and (NH_4_)_2_SO_4_ concentration were in the middle range. The optimized parameters predicted by RSM were 40 g/L of crude glycerol, 0.72 g/L of yeast extract, and 0.43 g/L of (NH_4_)_2_SO_4_ (Figure 2e). The lipid content and carotenoid concentration obtained from the experiment were found to be close to the predicted value. Similar results were observed for high carotenoid production in *R. paludigenum* DMKU3-LPK4 grown in 40-g/L glycerol [45]. Additionally, Uprety et al. reported that 40 g/L of pure glycerol was the optimum concentration for producing lipid content in *R. toruloides* ATCC 10788 [11]. Fermentation of *Trichosporandoides spathulata* JU4-57 was obtained at 13.8 g/L biomass with 32 g/L of crude glycerol using a 5 L stirred tank [46] (Table S1 [8,43,47,48]). These results indicated that RSM is a useful approach for optimizing the concentration of lipids and carotenoids in *R. paludigena* CM33. Therefore, the RSM results of 40 g/L of crude glycerol, 0.72 g/L of yeast extract, and 0.43 g/L of (NH_4_)_2_SO_4_ can be applied for upscaling in a 500 L fermenter.

### 2.3. Effects of Different Fermentations on Lipid and Carotenoid Productions Using a 500 L Fermenter

**Figure 3:**
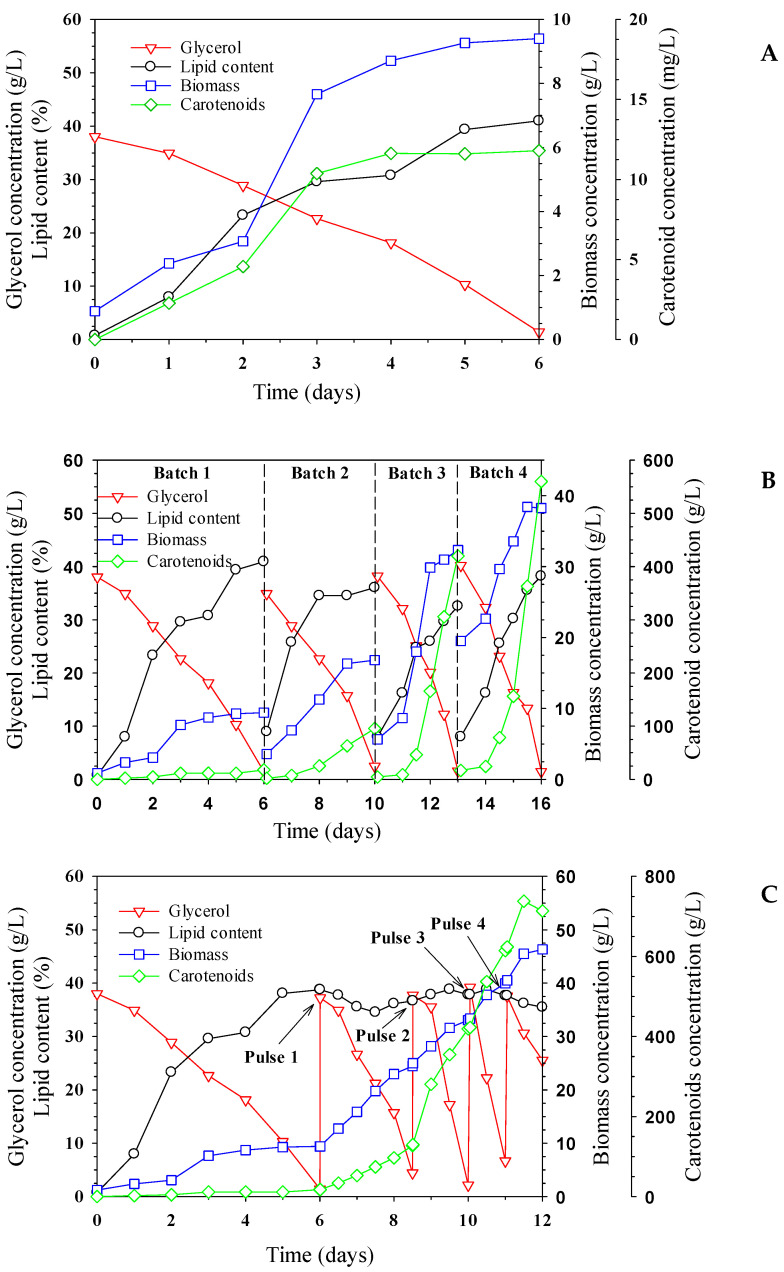
The effect of different fermentations on substrate consumption, cell growth, and products formation of *R. paludigena* CM33 in a 500 L fermenter: (**A**) batch, (**B**) repeated-batch, and (**C**) fed-batch mode. Legend: glycerol concentration (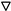), lipid content (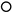), biomass concentration (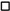), and carotenoid concentration (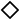), respectively.

The effects of different pilot-scale fermentations on lipid and carotenoid production are illustrated in Figure 3. Figure 3A shows the experimental result of batch fermentation with an initial substrate concentration of approximately 40 g/L. The result shows that the glycerol concentration constantly decreased from the beginning until it was completely consumed at the end of the fermentation process at 6 days. When the cells entered the exponential phase at 2 days after inoculation, the biomass concentration reached the maximum value of 9.27 g/L. The calculated biomass yield from the substrate was 0.23 g/g. The lipid content and carotenoid concentration were 40.99% and 11.82 mg/L, respectively. The improvement of fermentation efficiency was further investigated using a repeated-batch mode to increase productivity and reduce the operating time. Figure 3B shows the experimental results for a total of four batches within 16 days of fermentation. At the end of each batch, approximately 90% of the fermentation broth was removed, and the same volume of fresh sterile medium was added. It was observed that the initial biomass concentration increased with each batch, with a starting value of 0.89 g/L for the first batch, which increased to 3.56 g/L, 5.65 g/L, and 19.50 g/L for the second, third, and fourth batches, respectively. Higher initial biomass concentration resulted in higher productivity and a higher substrate-consumption rate. Compared to the batch fermentation result, the final biomass concentrations were 16.81 g/L, 32.34 g/L, and 38.25 g/L, representing increases of 0.78, 2.51, and 3.16-fold, respectively. The oil content of the cells remained constant in the range of 32–40%, and oil and carotenoid productivities primarily depended on the biomass concentration. Carotenoid concentrations reached 95.65 mg/L, 419.97 mg/L, and 559.98 mg/L, corresponding to increases of 4.38, 22.64, and 30.51-fold compared to batch fermentation, respectively. In addition, the fermentation time of each batch decreased from 6 days to 4 days and 3 days for the last 2 batches, indicating that the cost associated with the operating time could be decreased by up to 50%. The objective of the fed-batch strategy is to maximize the fermentation performance by the addition of a concentrated medium to avoid substrate-inhibition effects. The results of biomass concentration, lipid content, and carotenoid concentration for this strategy are presented in Figure 3C. A pulse-feeding strategy was investigated by adding a concentrated medium to reach 40 g/L of crude glycerol after the beginning of each pulse. At the end of the first pulse, the biomass concentration increased to 9.98 g/L, and the carotenoid concentration reached 33.53 mg/L. At the end of the second and third pulses, the biomass concentration increased to 15.38 g/L and 37.37 g/L, corresponding to increases of 1.65 and 4.03-fold compared to the results of batch fermentation, respectively. However, the addition of the fourth pulse resulted in a biomass concentration of 45.38 g/L, which was only a 15% increase, and a glycerol concentration of 25.5 g/L remained at the end of the fermentation. The decline in cell growth could probably contribute to the oxygen limitation in the broth. During cultivation with high cell density, sufficient oxygen supply is one of the most crucial parameters. Insufficient concentration of dissolved oxygen inhibits the TCA cycle, which limits intracellular NADH generation. Previous work has reported that high-cell-density cultivation of *R. glutinis* using oxygen-enriched air increased the biomass concentration from 110 g/L to 185 g/L in fed-batch mode [49]. In this work, the experiment could not be further investigated due to a lack of additional oxygen supply equipment. However, the carotenoid concentration obtained was 713 mg/L, which was the highest value compared to the other experiments. The fermentation performance of each process is summarized in Table 3. Compared to the batch process, the fed-batch fermentation strategy exhibited more than a 5-fold increase in biomass and a 12-fold increase in carotenoid production. Rodrigues reported that the carotenoid production of *R. mucilaginosa* cultivated in an agro-industrial medium also had a 12-fold increase in carotenoid production in a fed-batch fermentation setup compared to batch fermentation [40]. Moreover, the fed-batch co-culture of *R. glutinis* DBVPG 3853 and *Debaryomyces castellii* DBVPG 3503 with corn syrup as a carbon source resulted in a 150% increase in carotenoid production and a 2-fold increase in biomass compared to the batch culture [50]. Fed-batch culture supplemented with molasses resulted in a 4-fold increase in biomass compared to batch culture [51] (Table S1 [52,53,54]). Table S1 shows that various concentrations of crude glycerol were employed for cultivating *Rhodotorula* sp. This study demonstrates that using crude glycerol in conjunction with different pilot-scale fermentations increases biomass, lipid, and carotenoid production in comparison to batch fermentation. Moreover, scaling up the cultivation process contributes to an increase in high biomass density. Therefore, the results suggest that fed-batch cultivation using 40 g/L of crude glycerol, 0.72 g/L of yeast extract, and 0.43 g/L of (NH_4_)_2_SO_4_ can enhance the lipid content and carotenoid concentration in a 500 L fermenter at the pilot scale.

The analysis results of the cell composition in Table S2 indicate that yeast contains a reasonable nutritional content, including proteins (13.82 ± 0.04 g/100 g), total fat (43.21 ± 0.66 g/100 g), total carbohydrates (30.93 ± 0.95 g/100 g), moisture (10.71 ± 0.07 g/100 g), ash (2.97 ± 0.00 g/100 g), crude fiber (0.21 ± 0.02 g/100 g), and carotenoids (15.39 ± 0.04 mg/g of DCW). Numerous studies have been conducted to enhance carotenoid production in *Rhodotorula* sp. through the optimization of carotenoid fermentation conditions with the aim of maximizing carotenoid yield [52]. Moreover, based on our previous research, our findings suggest that when used as a probiotic supplement in shrimp feed, *R. paludigena* CM33 can enhance growth, bolster antimicrobial responses against VP_AHPND_, and improve flesh quality by increasing protein and lipid content [55]. This highlights its potential as a valuable supplement for shrimp diets and suggests its potential use as a supplement in animal feed. Similar to previous research, when cultivated with 25–100 g/L of crude and commercial glycerol, *R. glutinis* R4 accumulated lipids at rates of 44–57% and 41–52% (*w*/*w*), respectively [40]. Moreover, *Rhodotorula* is one of the most well-known types of oleaginous red yeasts and has the extraordinary capacity to synthesize a wide range of important carotenoids and lipids [42]. Thus, this study focused on *R. paludigena* CM33 and its role in these advancements and summarized its potential as an alternative source of natural bioproducts. Biotechnologically beneficial yeast strains have gained considerable attention recently due to their high demand across various industries.

### 2.4. Carotenoids Purification with High-Pressure Homogenization (HPH) and Preparative HPLC and the Analysis by HPLC, LC-MS, and NMR

**Figure 4:**
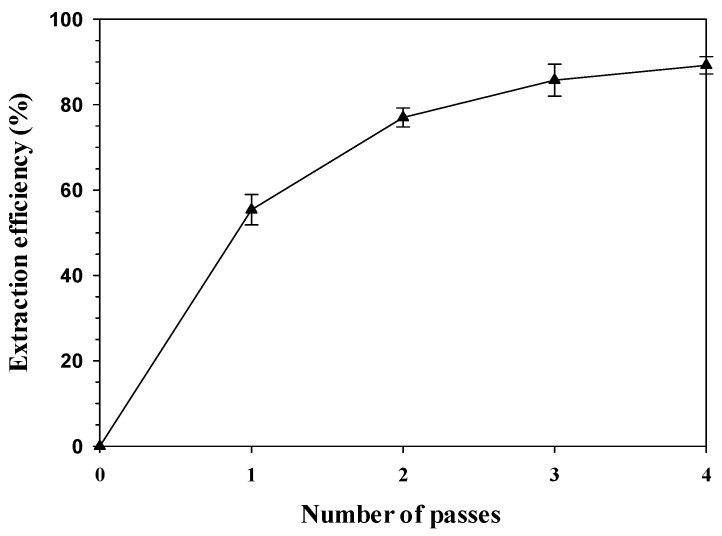
Extraction efficiency of carotenoids from *R. paludigena* CM33 suspensions as a function of passes. The pressure of HPH was operated at 30,000 psi with 5% cell concentration.

**Figure 5:**
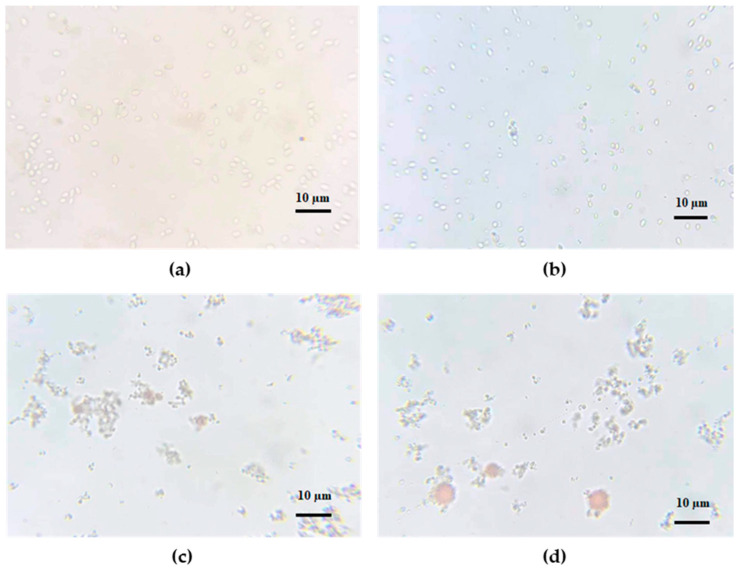
Microscopic images of *R. paludigena* CM33 cells (**a**) after the 1st pass, (**b**) after the 2nd pass, (**c**) after the 3rd pass, and (**d**) after the 4th pass using HPH.

The carotenoids were purified using high-pressure homogenization (HPH) and preparative HPLC, which are highly effective and scalable methods for extraction and product purification. HPH is particularly advantageous in providing efficient disruption and release of intracellular components [43,56], while preparative HPLC is a liquid chromatography technique that utilizes a preparative column with high loading and high resolution to achieve high-purity separation [47]. Figure 4 shows the extraction efficiency of carotenoids from *R. paludigena* CM33 suspensions as a function of passes. In this work, HPH was performed at a pressure of 30,000 psi with a 5% feed ratio to effectively disrupt the rigid cell walls. The resulting extraction efficiency was 55.42%, 76.99%, 85.75%, and 89.22% after 1–4 passes, respectively. The extraction efficiency of the fourth pass resulted in only a 4.05% increase compared to the third pass. This result suggested that the three passes is the optimum number of passes for this process. Figure 5a–d indicate that HPH caused complete cell disruption, as observed in the microscope images of cell disruption after the four passes. Samples are typically subjected to multiple passes through a high-pressure homogenizer to achieve the desired results [56]. This is similar to a previous study, where carotenoid extraction from *Sporidiobolus pararoseus* cells was conducted using HPH at a pressure of 80 MPa (11,603 psi) applied to an 8% biomass concentration with three passes [11]. Furthermore, HPH was explored as a cell-disruption method for the extraction of carotenoids from *Desmodesmus* sp. F51 [48].

**Figure 6:**
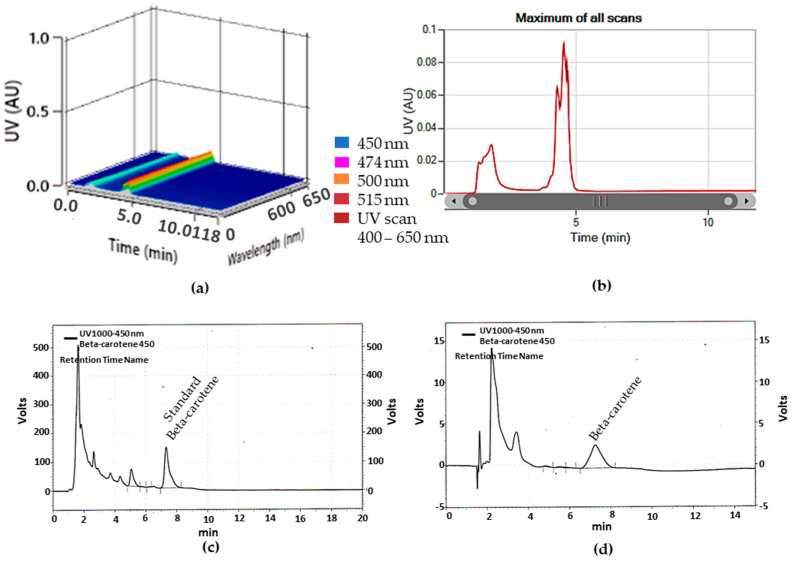
Preparative HPLC chromatogram of carotenoids from *R. paludigena* CM33; (**a**,**b**) beta-carotene and HPLC chromatogram of purified beta-carotene after preparative HPLC; (**c**) standard beta-carotene (**d**) beta-carotene sample after preparative HPLC was analyzed.

**Figure 7:**
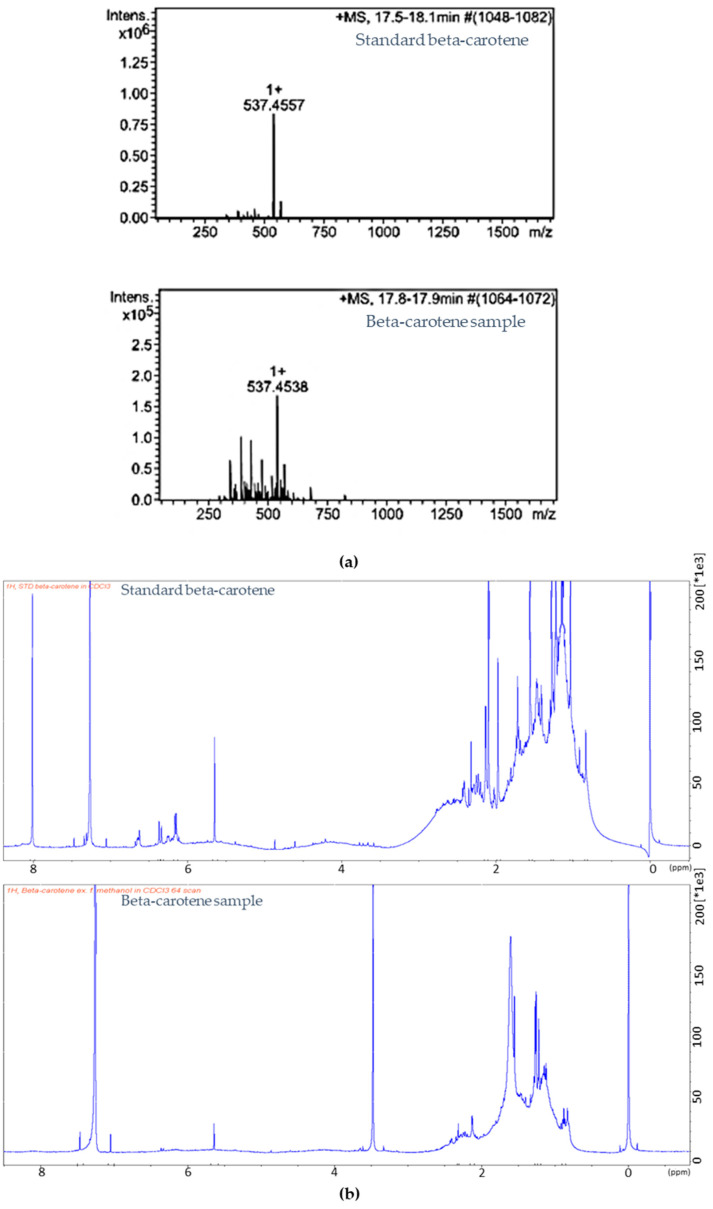
(**a**) Mass spectrum of beta-carotene standard and beta-carotene sample and (**b**) ^1^H NMR spectrum of beta-carotene standard and beta-carotene sample.

Optimal conditions for separating beta-carotene using preparative HPLC were established by optimizing parameters, including the mobile phase system (methanol:acetonitrile:ethyl acetate) and a flow rate of 30 mL/min. The detection mode was set at 450 nm, 474 nm, 500 nm, and 515 nm. The characteristics of the compound samples after the preparative HPLC process are shown in Figure 6b. The analysis determined that the observed compound corresponded to beta-carotene, with a retention time of 4.90 min (Figure 6a,b). Following preparative HPLC, the compound was compared to standard beta-carotene (Sigma, Saint Louis, MO, USA) dissolved in methanol. The analytical HPLC analysis demonstrated excellent resolution for beta-carotene, indicating a concentration of 12.76 mg/L and a purity of 94.80% compared to the retention time of the beta-carotene standard at 7.53 min (Figure 6d). In addition, both LC-MS and 1H NMR analyses were utilized to determine the structure of the purified beta-carotene compound. These analytical techniques serve as valuable tools for the characterization of carotenoids [53]. The LC-MS results displayed a molecular weight of 537.45 *m*/*z* for beta-carotene, as shown in Figure 7a, which was comparable to the molecular weight of the beta-carotene standard at 537.46 *m*/*z*. The 1H NMR analysis was conducted using a Bruker 500 MHz instrument to confirm the beta-carotene, as shown in Figure 7b. The molecular structure of the beta-carotene was successfully determined, as evidenced by the typical chromatogram pattern observed, which is indicative of beta-carotene. The resonance at 5 ppm in the 1H NMR spectrum corresponds to protons on a double bond, while the resonance of protons attached to methyl groups was observed between 1 and 2 ppm [54]. Additionally, the 1H NMR spectra of beta-carotene displayed resonances in the range of 1.5 to 2.5 ppm, which correspond to the protons attached to the ring (Figure 7b). The concentration of beta-carotene pigment synthesized from *R. glutinis* 32 accounted for more than 80% of the total carotenoids [51,57]. The results indicate that both preparative HPLC and cooperative HPH are effective methods for separating carotenoid structures from *R. paludigena* CM33 cells. The purified compound was confirmed to be beta-carotene through LC-MS and NMR analysis.

## 3. Materials and Methods

### 3.1. Microorganism and Culture Conditions

The oleaginous yeast *R. paludigena* CM33 was isolated from a natural habitat by our laboratory [4]. The yeast was grown on yeast peptone dextrose (YPD) medium (20 g/L glucose, 10 g/L peptone, 10 g/L yeast extract, and 15 g/L agar) [4]. The inoculum for fermentation was prepared using 10 mL of minimal medium components: 70 g/L glucose, 0.4 g/L KH_2_PO_4_, 0.55 g/L (NH_4_)_2_SO_4_, 2.0 g/L MgSO_4_·7H_2_O, and 0.75 g/L yeast extract [2]. It was then incubated at 30 °C for two days on rotary shaker at 150 rpm for 48 h [4].

### 3.2. Characteristics of Chemical Composition and Elemental Analysis of Crude Glycerol

Crude glycerol (BioSynergy, Nakhon Ratchasima, Thailand) was quantitatively analyzed using GC-MS (Agilent Technologies 7000B, Santo Clara, CA, USA). The free glycerol content of crude glycerol was determined using GC analysis as follows: 40–100 mg of weighed crude glycerol was acidified with 100 µL of 1:1 HCl (*v*/*v*) and dissolved in 10 mL of pyridine in a 15 mL glass test tube. The resulting solution was mixed with 100 µL of a 1,2,4-butanetriol standard solution (0.89 mg/mL, internal standard) and derivatized with MSTFA (100 µL) at 38 °C for 15 min. After filtration, the sample was injected into an MXT Biodiesel TG column (14 m, 0.53 mm, 0.16 μm) at a volume of 1 μL. Helium was used as the carrier gas at a flow rate of 3 mL/min. The detector temperature was maintained at 380 °C, while the injector and column temperatures were ramped from 50 to 110 °C at a rate of 5 °C/min. A calibration curve was constructed by analyzing pure glycerol at various concentrations [36]. The pH was determined by a pH meter (Oakton pH700 Illinois, USA). The carbon, hydrogen, nitrogen, and sulfur concentrations of crude glycerol were determined by a CHNS/O (liquid sample) analyzer (LECO CHN628 + TruSpecMicro, St. Joseph, MI, USA), and Na, Mg, Al, P, K, Ca, Mn, Fe, Co, Cu, and Zn were analyzed using an inductively coupled plasma optical emission spectrometer (ICP-OES) (Perkin Elmer Optima 8000, Waltham, MA, USA). Crude glycerol (1–5 g) was digested using 10 mL of trace metal grade HNO_3_ in a microwave digester (METASH, MWD Series, Matthews, NC, USA). The digester temperature was ramped to 200 °C after 15 min, maintained at 200 °C for 15 min, and cooled down to 25 °C. The digested crude glycerol solution was transferred into a 500 mL volumetric flask. The solution was mixed and analyzed using ICP-OES [36]. As pretreatment for the crude glycerol, it was adjusted with 37% HCl to pH 1.70 ± 0.01. It was then mixed thoroughly using a magnetic stirrer for 1 h and left undisturbed for an additional 12 h. This resulted in the separation of the crude glycerol into three layers, with the top layer containing fatty acids, the middle layer containing glycerol, and the bottom layer containing inorganic salt precipitates. Next, the glycerol from the middle layer was further adjusted to pH 6.0 using 10 N NaOH for 30 min [58]. The clear upper portion was used for the experiment, and the glycerol concentration was analyzed using HPLC (Hitachi Chromaster 5110 Tokyo, Japan) with an HPX-87C column (7.8 mm × 150 mm i.d., 9 μm) and a refractive index (RI) detector (Model 5450 RI detector Tokyo, Japan). For the analysis of the samples, 5 mM sulfuric acid was used as the mobile phase, and the flow rate was maintained at 0.6 mL/min [8].

### 3.3. Response Surface Methodology (RSM)—Box–Behnken Designs

The optimization of *R. paludigena* CM33 cultivation was studied using Box–Behnken designs. The effects of the optimal conditions on the carotenoid concentration and lipid content were studied through the Box–Behnken method using Design Expert 13 (Stat-Ease Inc., Minneapolis, MN, USA). The independent variables and their levels are shown in Table S3. The Box–Behnken design consisted of 15 factorial points, as given in Table S4. The predicted and obtained responses regarding carotenoid and lipid production were analyzed. The response variables could be fitted to the general form of a quadratic polynomial model shown in Equation (3).

(3) $${y=\beta }_{0}+\overset{3}{\underset{i=1}{\sum }}\beta_{i}x_{i}+\overset{3}{\underset{i=1}{\sum }}\beta_{\mathrm{ii}}x_{i}^{2}{+\Sigma }_{i=1}^{2}\overset{3}{\underset{j=i+1}{\sum }}\beta_{\mathrm{ii}}x_{i}x_{j}$$

where y is the response variable measured for each combination of factorial level; ꞵ_0_, ꞵ_i_, ꞵ_ii_, and ꞵ_ij_ are regression coefficients for the linearity, intercept, interaction, and square; and x_i_ and x_j_ represent the independent variables.

### 3.4. Cultivation of R. paludigena CM33 Using a 500 L Fermenter

The seed culture was prepared as follows. *R. paludigena* CM33 was inoculated into crude glycerol medium and separately cultivated in 5 L and 50 L fermenters for 48 h at 30 °C and 0.7 vvm. To conduct batch fermentation, 10% (*v*/*v*) cultures were inoculated into 300 L of fermentation medium and cultivated for 7 days at 30 °C with air flow of 0.7 vvm. For the repeated-batch fermentation, 300 L of the fermentation broth was removed once the total glycerol concentration was below 10 g/L. An equal volume of fresh medium was then sterilized in a separated pressurized tank. After cooling, the fresh medium was aseptically introduced into the 500 L fermenter. The entire repeated-batch fermentation process consisted of 4 batches. For the fed-batch fermentation with pulse addition, approximately 10% (*v*/*v*) of cultures were inoculated into 250 L of media and cultivated at 30 °C with air flow of 0.7 vvm. The fed-batch culture was conducted in four stages. During the first stage, 25 L of the concentrate medium was added to the fermenter, reaching a total glycerol concentration of 40 g/L. This addition occurred when the residual total glycerol concentration dropped below 5 g/L after 3 days. Subsequently, in the second to fourth stages, 25 L of the medium was added each time to maintain a constant total glycerol concentration of 40 g/L. Throughout the experiment, a 25 mL sample of the fermentation broth was collected daily to measure carotenoid concentration, lipid content, OD 600 nm, biomass, and glycerol concentration. Carotenoid measurement was conducted using a spectrophotometer [59]. The individual fermentation cycles were established using a previously reported method [60]. The pH was maintained at 5.5 ± 0.5 using 10 N NaOH. Silicone antifoam (Kemaus, Australia) was used to control foam formation as needed.

After fermentation, yeast cells were harvested by centrifugation (Avanti JXN-26, Beckman Coulter, Brea, CA, USA) at 3500× *g* for 10 min and washed twice with 500 mL of deionized water, discarding the supernatant each time. The cells were then freeze-dried (Freeze Dry, Christ/Gamma2-16, Osterode am Harz, Germany) [61] and stored at a temperature of −20 °C until utilized. To analyze the nutritional components, the yeast samples underwent proximate analytical procedures following the guidelines of the Association of Official Analytical Chemists (AOAC) [62]. These methodologies identified six dietary components: total carbohydrates, protein, total fat, moisture, ash, and crude fiber. The total carotenoids were analyzed using the method by Ribeiro et al. [59]. Figure S1 shows the experimental setup for fermentations in the 500 L fermenter (A), an agar plate of *R. paludigena* CM33 (B), and the sample of lyophilized *R. paludigena* CM33 (C).

### 3.5. Carotenoids Purification with High-Pressure Homogenizer and Preparative High-Performance Liquid Chromatography

Cell suspensions were treated with a high-pressure homogenization (HPH) technique using a microfluidizer^®^ processor (Microfluidics Model M-110EH-30, Westwood, MA, USA) at a constant pressure of 30,000 psi and 5% cell concentration [63]. The extraction efficiencies of carotenoids obtained with 1 to 4 passes were compared. After each pass, samples were centrifuged at 5000 rpm for 10 min to separate pellets and supernatant. The extracted carotenoid was then analyzed for carotenoid concentration while the pellets were washed with deionized water, and the degree of cell disruption was evaluated using a microscope. The extraction efficiency was calculated from the ratio of carotenoid yield from HPH to the extraction yield with chloroform and methanol (2:1 *v*/*v*)

Preparative HPLC (BÜCHI Pure Flash/Prep HPLC Model C-850, Flawil, Switzerland) was used to separate and purify the carotenoids extracted from *R. paludigena* CM33. The mobile phase consisted of methanol, acetonitrile, and ethyl acetate (76:12:12 *v*/*v*) [64]. The separation was dissolved in 100 mL of mobile phase. The sample was injected into the HPLC system equipped with an EcoFlex C18 12 g column with 50 µm spherical particles. The flow rate was 30 mL/min, and the analysis wavelengths were 450, 474, 500, and 515 nm. After purification, the carotenoids were confirmed by HPLC, LC-MS, and NMR.

### 3.6. Analytical Methods

Carotenoid extraction was carried out according to Ribeiro et al. [59]. Briefly, cells were harvested by centrifugation at 12,000× *g* for 5 min (Denville Microcentrifuge 260D New Jersey, USA) and resuspended with 1 mL of deionized water. After discarding the supernatant, the cell pellets were frozen at −20 °C for 24 h. The frozen pellets were then thawed and disrupted before being resuspended in 2 mL of DMSO. The resulting mixture was vortexed for 2 min and incubated at 60 °C for 15 min. Next, 2 mL of acetone, 2 mL of petroleum ether, and 2 mL of 20% NaCl were sequentially added to the mixture. The entire mixture was vortexed for a total of 5 min and then centrifuged at 12,000× *g* for 5 min. The carotenoid concentration was measured by a spectrophotometer (Thermo Scientific P1000 UV-Vis Wisconsin, USA) at a wavelength of 450 nm using the absorption coefficient of beta-carotene in PE (A1%1 cm = 2592) [59].

The optical density (OD) was measured at 600 nm using a spectrophotometer (Thermo Scientific Genesys10S UV-Vis Wisconsin, Waltham, MA, USA), and the OD of the medium was subtracted for each sample [65]. For biomass, a sample of 2 mL was centrifuged at 12,000× *g* for 2 min, the supernatant was discarded, and cells were washed with deionized water three times and dried at 65 °C for 24 h to constant weight [24]. For lipid content (%), 300 mg of cells (dry weight) was ground into powder and mixed with 50 mL of chloroform and methanol (2:1) incubated at 30 °C overnight on a rotary shaker at 150 rpm. Samples were mixed with 10 mL of deionized water and incubated at room temperature until the solution separated into two phases. The low phases were collected and evaporated to obtain lipids, and samples were weighed until the weight was stable [66].

Glycerol was determined quantitatively by HPLC (Hitachi Chromaster 5110 Tokyo, Japan) using an HPX-87C column (7.8 mm × 150 mm i.d., 9 μm) with an RI detector (Model 5450 RI detector Tokyo, Japan). For the analysis of the samples, 5 mM sulfuric acid was used as the mobile phase, and the flow rate was maintained at 0.6 mL/min [8].

A SpectraSystem (Thermo Scientific P1000 UV-Vis Wisconsin, USA) equipped with a SuperC18 HPLC column (4.6 mm × 150 mm i.d., 5 μm) was used for HPLC analysis. The detection conditions were optimized before analysis. The mobile phase consisted of a mixture of methanol, acetonitrile, and water at a ratio of 72:12:12 [64]. The flow rate was set to 1 mL/min, the column temperature was maintained at 45 °C, the injection volume was 10 μL, and the analysis wavelength was set to 450 nm [67]. The concentration of the beta-carotene pigment was determined by referencing a standard curve for beta-carotene. The identification of beta-carotene in the compound was based on its relative retention times compared to the standard beta-carotene (MilliporeSigma, Burlington, MA, USA) [68].

Liquid chromatography–mass spectrometry (LC-MS) was used to identify different carotenoids with a UHPLC Ultimate 3000 liquid chromatograph (Thermo Fisher Scientific, Waltham, MA, USA) coupled with a Bruker/micrOTOF–Q II mass spectrometer. An Agilent InfinityLab Poroshell 120 pentafluorophenyl (PFP) column (4.6 mm × 100 mm, 2.7 µm) was used for separation. The mass spectra of carotenoids were acquired with an *m*/*z* detector in the scan ranges of 50–1700 *m*/*z* and 50–1700 by a diode array detector, and the results were confirmed with the respective standards. The LC-MS analysis of carotenoids was conducted in positive ion mode (APCI) with a capillary voltage of 4500 V for the total ion current (TIC). Nitrogen was used as the nebulizer gas (purity 99.9%) with a pressure of 0.3 Bar and a vaporizer temperature of 180 °C [53].

^1^H Nuclear Magnetic Resonance Spectroscopy (NMR) measurements were performed on a Bruker AVANCE III 500 MHz spectroscope (Bruker, Billerica, MA, USA) at 500 MHz. Purified compounds were dissolved in methanol and acetone, and data were processed by Bruker Topspin 3.5 software [34].

## 4. Conclusions

This study investigated the use of crude glycerol as a substrate for intracellular lipid and carotenoid production. RSM was successfully used to investigate the production of carotenoids and lipids. The results revealed that the optimum media composition was 40 g/L of crude glycerol, 0.72 g/L of yeast extract, and 0.43 g/L of (NH_4_)_2_SO_4_ in a 500 L fermenter with fed-batch fermentation, which led to increased cell density, lipid, and carotenoid production. High-efficiency carotenoid extraction was achieved using HPH. Using preparative HPLC, high-purity beta-carotene was obtained. The results indicate that crude glycerol can be a viable alternative substrate to increase carotenoid and lipid production by *R. paludigena* CM33. Further research is being performed to explore the extended applications of carotenoids and lipids produced by *R. paludigena* CM33, including antioxidant testing, anti-inflammatory properties, and the discovery of new functionalities. This investigation reveals the potential for these yeast-based carotenoids to be transformed into a valuable commodity, particularly for the industrial manufacturing of bioactive products.

## Figures and Tables

**Table 1:** Chemical composition and elemental analysis of crude glycerol.

Parameter	Crude Glycerol	Unit
Free glycerol	58	%
pH	2.38	-
C	25.909	%
H	7.440	%
N	<1	%
S	4.139	%
Na	11,769 ± 0.10	ppm
Mg	31.29 ± 0.12	ppm
Al	ND	ppm
P	38.7 ± 0.50	ppm
K	118.8 ± 0.13	ppm
Ca	2050.080 ± 0.14	ppm
Mn	3.22 ± 0.42	ppm
Fe	31.6 ± 0.34	ppm
Co	ND	ppm
Cu	4.960 ± 0.14	ppm
Zn	25.79 ± 0.31	ppm

**Table 2:** Analysis of variance (ANOVA) and significance level of the response surface linear model exhibiting carotenoid concentration and lipid content.

Source	Sum of Squares	Degree of Freedom	Mean Square	F-Value	*p*-Value	
**Carotenoid concentration (ug/L)**						
**Model**	257,800	9	28,639.59	216.78	<0.0001	significant
**A**	79,600.50	1	79,600.50	602.51	<0.0001	significant
**B**	798.47	1	798.47	6.04	0.0573	not significant
**C**	21.90	1	21.90	0.1657	0.7008	not significant
**Residual**	660.58	5	132.12	–	–	
**Lack of Fit**	454.58	3	151.53	1.47	0.4291	not significant
**Pure Error**	206.00	2		–	–	
**Cor Total**	258400	14	–	–	–	
R^2^ = 0.9974; R^2^_adj_ = 0.9928						
**Lipid content (%)**						
**Model**	4570.69	9	507.85	104.44	<0.0001	significant
**A**	1891.13	1	1891.13	388.89	<0.0001	significant
**B**	50.79	1	50.79	10.44	0.0232	significant
**C**	1.11	1	1.11	0.2277	0.6534	not significant
**Residual**	24.31	5	4.86	–	–	
**Lack of Fit**	20.54	3	6.85	3.63	0.2234	not significant
**Pure Error**	3.77	2		–	–	
**Cor Total**	4595.00	14	–	–	–	
R^2^ = 0.9947; R^2^_adj_ = 0.9852						

**Table 3:** Summary of carotenoid concentrations and lipid accumulation in *R. paludigena* CM33 cultivation under different fermentation processes.

Processes	Volume	OD (600 nm)	Biomass (g/L)	Lipid Content (%)	Carotenoids (mg/g CDW)	Carotenoids (mg/L)
Batch	350 L	15.94	9.27	40.99	1.27	11.80
Repeated-batch	350 L					
	Batch 1	19.35	9.40	40.95	1.89	17.77
	Batch 2	27.92	16.81	36.04	5.69	95.65
	Batch 3	63.72	32.33	32.55	12.99	419.97
	Batch 4	71.76	38.25	38.22	14.64	559.98
	Total 1330 L		$\bar{x}$ = 24.20	$\bar{x}$ = 36.94	$\bar{x}$ = 8.80	$\bar{x}$ = 273.34
Fed-batch	350 L					
	1st pulse	16.54	9.98	12.08	3.36	33.53
	2nd pulse	25.54	15.38	44.48	5.20	79.98
	3rd pulse	62.12	37.37	26.73	12.67	473.48
	4th pulse	75.45	45.38	38.78	15.39	698.40

## Data Availability

Data are available upon request to the corresponding author.

## Supplementary Materials

The following supporting information can be downloaded at: https://www.mdpi.com/article/10.3390/ijms242417192/s1.
