# Supplementary material for: Simultaneous Lipid and Carotenoid Production via Rhodotorula paludigena CM33 Using Crude Glycerol as the Main Substrate: Pilot-Scale Experiments
Source: Int J Mol Sci. 2023 Dec 6;24(24):17192. doi: 10.3390/ijms242417192 (PMC10743220; doi:10.3390/ijms242417192)
Supplement: Supplementary file 1 [file ijms-24-17192-s001.zip › ijms-2661983-supplementary.pdf]

Supplementary data

Simultaneous Lipid and Carotenoids Production by *Rhodotorula paludigena* CM33 using Crude Glycerol as the Main Substrate: Pilot-Scale Experiments

Cheeranan Sriphuttha [1], Pailin Boontawan [1], Pasama Boonyanan [3], Mariena Ketudat-Cairns [1] and Apichat Boontawan[1,2,*]

[1] School of Biotechnology, Institute of Agricultural Technology, Suranaree University of Technology, 111 University Avenue, Muang district, Nakhon Ratchasima, THAILAND 30000

[2] Center of Excellent in Agricultural Product Innovation, Suranaree University of Technology, 111 University Avenue, Muang district, Nakhon Ratchasima, THAILAND 30000

[3] Technopolis, Suranaree University of Technology, 111 University avenue, Muang district, Nakhon Ratchasima, THAILAND 30000

* Correspondence: apichat@sut.ac.th; Tel.: +66-44-224234

Table S1. The cultivation of yeast on different substrates was observed in terms of lipid, biomass, and carotenoid production.

Strain	Substrate	Operating conditions	Cultivation method	Lipid content	Carotenoids	Biomass	Reference
R. paludigenum DMKU3-LPK4	40 g/L glycerol	Incubated in a shake flash at 30 °C, pH 6.0 and 130 rpm	Batch	-	3.42 mg/L	7.59 g/L	[45]
R. toruloides ATCC 10788	40 g/L pure glycerol	Incubated in a shake flash at 30 °C, pH 6.0, 200 rpm, KH_2PO_4 1.0, $Na_2HPO_4{\cdot}12H_2O$ 1.0, $MgCl_2{\cdot}6H_2O$ 1.5, pH 6.0	Batch	53.28%	-	21.16 g/L	[8]
T. spathulata JU4-57	40% crude glycerol	Incubated in a 5-L Stirred Tank at 30 °C, pH 6.0 and aeration rate at 1–3 vvm	Fed-batch	56.40%	-	13.80 g/L	[46]
R. mucilaginosa CCT 7688	70 g/L sugarcane molasses and 3.4 g/L corn steep liquor	Incubated in a 250 mL Erlenmeyer flash at 25 °C and 180 rpm	Batch	-	1.25 mg/L	7.90 g/L	[40]
R. mucilaginosa CCT 7688	30 g/L sugarcane molasses and 6.5 g/L corn steep liquor	Incubated in a 500 mL Erlenmeyer flash at 25 °C and 180 rpm	Fed-batch	-	3.73 mg/L	16 g/L	[40]
R.glutinis Rh-00301	400 g/L glycerol	Incubated in 5 L bioreactors at 30 °C, pH 5.5 and aeration rate at 1 vvm	Fed-batch	63 ± 6%	-	106 g/L	[49]
R. glutinis DBVPG 3853 and *D. castellii* DBVPG 3503	8.3 g/L glucose, 22.7 g/L maltose, and 4.65 g/L nitrogen	Incubated in 1 L bioreactors at 30 °C, and 220 rpm	Fed-batch	-	8.20 mg/L	-	[50]
R. paludigena CM33	40 g/L crude glycerol	Incubated in a 500 L fermenter at 30 °C, pH 6.0 and flow level 0.7 vvm	Batch	40.99%	1.27 mg/g	9.27 g/L	This work
R. paludigena CM33	40 g/L crude glycerol	Incubated in a 500 L fermenter at 30 °C, pH 6.0 and flow level 0.7 vvm	Repeated batch	36.04%	14.64 mg/g	38.33 g/L	This work
R. paludigena CM33	40 g/L crude glycerol	Incubated in a 500 L fermenter at 30 °C, pH 6.0 and flow level 0.7 vvm	Fed-batch	44.48%	15.39 mg/g	45.38 g/L	This work

Table S2. Proximate compositions of *R. paludigena* CM33.

Parameters	*R. paludigena* CM33
	Freeze-dried cells
Moisture (g/100g)	10.71±0.07
Protein (g/100g)	13.82±0.04
Total fat (g/100g)	43.21±0.66
Total carbohydrate (g/100g)	30.93±0.95
Ash (g/100g)	2.97±0.00
Crude fiber (g/100g)	0.21±0.02
Energy (Kcal/100g)	567.87±2.46
Carotenoids (mg/g)	15.39±0.04
β-carotene (mg/g)	10.16±0.08
Torulene (mg/g)	3.25±0.01
Other	0.86±0.00

Table S3. Evaluated factors, factor notation, and their levels in Box-Behnken.

Independent variables	codes	Factor levels		
		-1	0	1
Crude glycerol (g/L)	A (X_1)	20	40	80
Yeast extract (g/L)	B (X_2)	0.50	0.63	0.75
$(NH_4)_2SO_4$ (g/L)	C (X_3)	0.25	0.40	0.55

Table S4. Box-Behnken designs consisting of 15 experiments for three experimental factors for the carotenoid concentration and lipid content by *R.paludigena* CM33.

Run	Crude glycerol (g/L)	Yeast extract (g/L)	$(NH_4)_2SO_4$ (g/L)	Carotenoid concentration (ug/g)		Lipid content (%)	
	Independent variables			Dependent variables			
				observed	predicted	observed	predicted
1	20	0.75	0.40	206.00	214.00	37.55	36.87
2	40	0.50	0.25	287.00	295.45	38.37	37.84
3	40	0.75	0.55	332.00	323.55	45.46	45.99
4	40	0.63	0.40	339.00	328.00	42.55	42.44
5	40	0.63	0.40	319.00	328.00	41.02	42.44
6	80	0.63	0.25	0	0.37	0	-0.60
7	20	0.63	0.55	200.00	203.26	30.94	30.92
8	40	0.50	0.55	298.00	295.56	35.90	37.23
9	40	0.63	0.40	326.00	328.00	43.76	42.44
10	80	0.63	0.55	0	7.63	0	-1.84
11	80	0.75	0.40	0	-2.00	0	1.49
12	80	0.50	0.40	0	-6.00	0	0.95
13	40	0.75	0.25	317.00	319.45	43.85	42.52
14	20	0.50	0.40	177.00	177.00	28.82	27.07
15	20	0.63	0.25	215.00	203.74	25.69	28.15

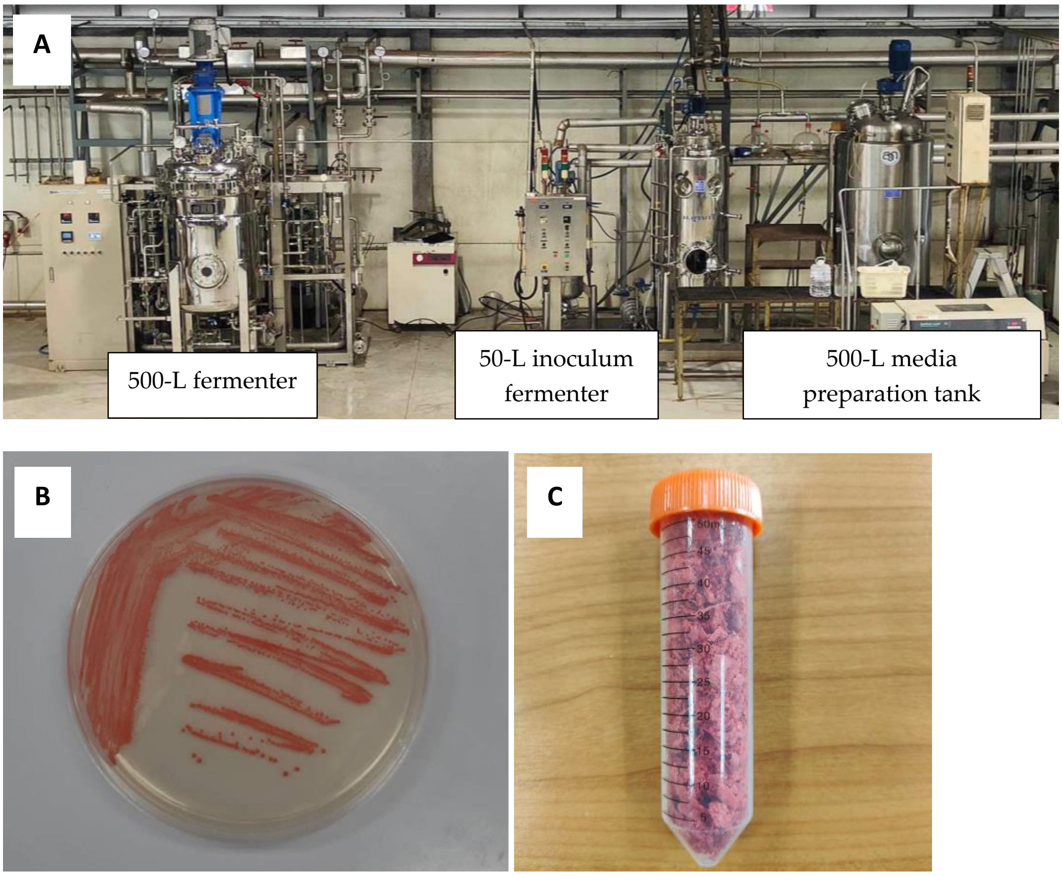

Figure S1. Experimental setup for the 500-L fermenter, 50-L inoculum fermenter, and 500-L media preparation tank (A), agar plate of *R. paludigena* CM33 (B), and the sample of lyophilized *R. paludigena* CM33 after freeze drying (C).
